# Developmental and adult acclimation impact cold and drought survival of invasive tropical *Drosophila kikkawai*

**DOI:** 10.1242/bio.058527

**Published:** 2021-06-08

**Authors:** Ravi Parkash, Chanderkala Lambhod, Ankita Pathak

**Affiliations:** Department of Genetics, Maharshi Dayanand University, Rohtak 124001, India

**Keywords:** Body color morphs, Developmental and adult acclimation, Stress resistance traits, Energy metabolites, Invasive tropical *Drosophila kikkawai*

## Abstract

Narrow distribution patterns of tropical *Drosophila* species are limited by lower resistance to cold or drought. In the invasive tropical *Drosophila kikkawai*, we tested whether developmental and adult acclimations at cooler temperatures could enhance its stress resistance level. Adult acclimation of winter collected body color morphs revealed a significant increase in the level of cold resistance. For light morph, its abundance during winter is not consistent with thermal-melanism hypothesis. However, higher cold acclimation capacity, as well as storage of energy metabolites could support its winter survival. In the wild-caught light and intermediate morphs, there is a lack of trade-off between cold and heat resistance but not in the case of dark morph. Developmental plasticity (15°C) resulted in the fivefold increase of cold survival at 0°C; and a twofold increase in desiccation resistance but a modest reduction (∼28–35%) in heat resistance as compared to morph strains reared at 25°C. Drought acclimation changes were significantly higher as compared with cold or heat pretreatment. We observed a trade-off between basal resistance and acclimation capacity for cold, heat, or drought resistance. For homeostatic energy balance, adult acclimation responses (cold versus drought; heat versus drought) caused compensatory plastic changes in the levels of proline or trehalose (shared patterns) but different patterns for total body lipids. In contrast, rapid cold or heat hardening-induced changes in energy metabolites were different as compared to acclimation. The ability of *D. kikkawai* to significantly increase stress tolerance through plasticity is likely to support its invasion potential.

## Abstract (precis)

**Summary:** In body color morphs of tropical *Drosophila kikkawai*, plasticity induced a higher level of resistance to cold and drought as well as three energy metabolites, which are likely to support its invasive potential.

## INTRODUCTION

In the genus *Drosophila*, distribution patterns of generalist, cold, or warm adapted species match their adaptations to stressful climatic conditions across seasons or geographical localities on different continents (Hoffmann and Parsons, 1997; Hoffmann et al., 2003; Hoffmann and Weeks, 2007). Cosmopolitan *Drosophila* species have a high level of resistance to climatic stressors (cold, heat, or drought) and therefore occur abundantly in all the six bio-geographical regions (Hoffmann and Parsons, 1991; Hoffmann, 2010). In contrast, restricted distribution of tropical *Drosophila* species is consistent with their lower physiological tolerance to cold and drier environments (Hoffmann et al., 2003; Kellermann et al., 2009; Hoffmann, 2010). Despite the cold sensitivity of tropical drosophilids, few Drosophila species (*Drosophila malerkotliana*, *D. kikkawai*, *Drosophila suzukii*, *Drosophila nasuta*) are able to extend their distribution range and invade new territories during the last few decades (Yuzuki and Tidon, 2020). For some tropical *Drosophila* species, higher resistance to cold and drought is evident when reared under lower growth temperature i.e. *D. malerkotliana* (Parkash et al., 2013) and *D. suzukii* (Shearer et al., 2016). Other tropical *Drosophila* species (*D. kikkawai*, *D. nasuta*) are likely to expand their distribution range due to increase in their tolerance levels to cold and drought (as a consequence of developmental plasticity and adult acclimation). However, no previous study has assessed stress resistance traits in *D. kikkawai* flies reared at cooler temperatures (Parkash and Ranga, 2013; Ranga et al., 2017).

In insects, genetic and plastic changes in cuticular traits (body melanization and cuticular lipids) are associated with tolerance to seasonally varying cold or drought conditions (Hadley, 1994; Majerus, 1998; Kalra et al., 2014). In drosophilids, there are different patterns of genetic control (monogenic versus polygenic) of abdominal melanization (Wittkopp et al., 2003). For example, in *Drosophila melanogaster*, melanization patterns show continuous quantitative variation within populations; and exhibit a gradient (cline) across geographical populations (Wittkopp et al., 2003; Parkash et al., 2008). In contrast, some *Drosophila* species of *montium* species subgroup show discrete darker or lighter morphs consistent with Mendelian inheritance (Ohnishi and Watanabe, 1985; Wittkopp et al., 2003). A characteristic feature of *D. kikkawai* is the occurrence of genetically controlled body color morphs (dark and light) limited to females (Parshad and Paika, 1964; Bock, 1980; Ohnishi and Watanabe, 1985). In drosophilids, body-color changes are either associated with genetic polymorphism or with phenotypic plasticity (Wittkopp et al., 2003). Two previous studies have reported a lack of developmental plasticity in body color morphs in two non-invasive *Drosophila* species, i.e. in *Drosophila falleni* (Dombeck and Jaenike, 2004) and *Drosophila polymorpha* (Brisson et al., 2005). However, the invasion potential of *D. kikkawai* on other continents may involve plasticity as well as genetic polymorphism of body color morph strains affecting tolerance to colder and drier climatic conditions

In the cosmopolitan *D. melanogaster*, several studies have shown increased levels of cold tolerance through developmental plasticity and adult acclimation (Hoffmann et al., 2003; Rako and Hoffmann, 2006; Colinet and Hoffmann, 2012). However, comparative analysis of cosmopolitan and tropical drosophilids have revealed mixed responses for plastic changes (adult hardening/acclimation) in different stress resistance traits (Sørensen et al., 2016). Rapid drought hardening of tropical and widely distributed *Drosophila* species (reared at 25°C) revealed a trade-off between basal and induced levels of desiccation resistance (Hoffmann, 1991; Kellermann et al., 2018). Cold or heat hardening capacity of 18 *Drosophila* species showed contrasting patterns, i.e. negative correlation between basal and induced cold tolerance but a positive correlation for heat tolerance (Nyamukondiwa et al., 2011). Thus, among drosophilids, plasticity levels for stress resistance vary across traits, species, and populations. In *D. melanogaster*, cold, heat or drought conditions impact perturbations in the level of energy metabolites (Djawdan et al., 1998; Overgaard et al., 2007; Michaud et al., 2008). In tropical invasive *Drosophila* species, genetically determined body color morphs are likely to differ in the levels of storage and utilization patterns for different energy metabolites but this aspect has received less attention so far.

*Drosophila kikkawai* belongs to the *montium* species subgroup of the subgenus Sophophora (Bock, 1980; Markow and O'Grady, 2006). *Drosophila kikkawai* is a chill-susceptible species and has not been recorded from colder environments in the temperate regions (Bock, 1980; Ashburner et al., 2003; Markow and O'Grady, 2006). Previous studies on latitudinal populations of *D. kikkawai* (reared at 21°C) have shown clinal variation for desiccation related traits (Parkash and Ranga, 2013); as well as for heat-resistance in *D. kikkawai* and its sibling *Drosophila leontia* (Ranga et al., 2017). *Drosophila kikkawai* is a non-pest species and its invasion potential likely depends upon its ability to increase survival under colder and drier climatic conditions. In the present work, we examined seasonal changes in the frequency of body color morphs of wild-collected *D. kikkawai* during three years. Isolation of true breeding strains for dark and light body color morphs and testing their developmental plasticity resulted in finding an intermediate strain (due to changes in melanization across 15 versus 25°C) while there was reduced plasticity in the dark and light morph strains. In winter collected body color morphs, we examined adult hardening or acclimation effects on chill-coma recovery time and heat knockdown; and compared relative acclimation capacity for each trait as well as morph. We investigated developmental plastic changes in resistance to cold, heat, and drought in the body color morph strains (reared under constant 15°C or 25°C with 50% relative humidity as a proxy of winter or spring seasons). Besides developmental acclimation, we tested acclimation of adult flies of each morph to cold or heat or drought pretreatments. We compared relative acclimation capacity for developmental plasticity as well as adult acclimation effects. We also tested stress resistance to cold or heat; and desiccation related traits (hydration, dehydration tolerance and cuticular traits) in body color morph strains reared under low versus high humidity conditions at 25°C. Finally, we compared plastic changes in three energy metabolites (trehalose, proline and body lipids) in response to rapid hardening or acclimation to cold or heat or low humidity stress in body color strains reared at 15°C. Thus, we assessed whether plasticity can improve cold or heat or drought tolerance of this tropical *Drosophila* species.

## RESULTS

### Seasonal changes in the frequency of body color morphs

**Fig. 1.**
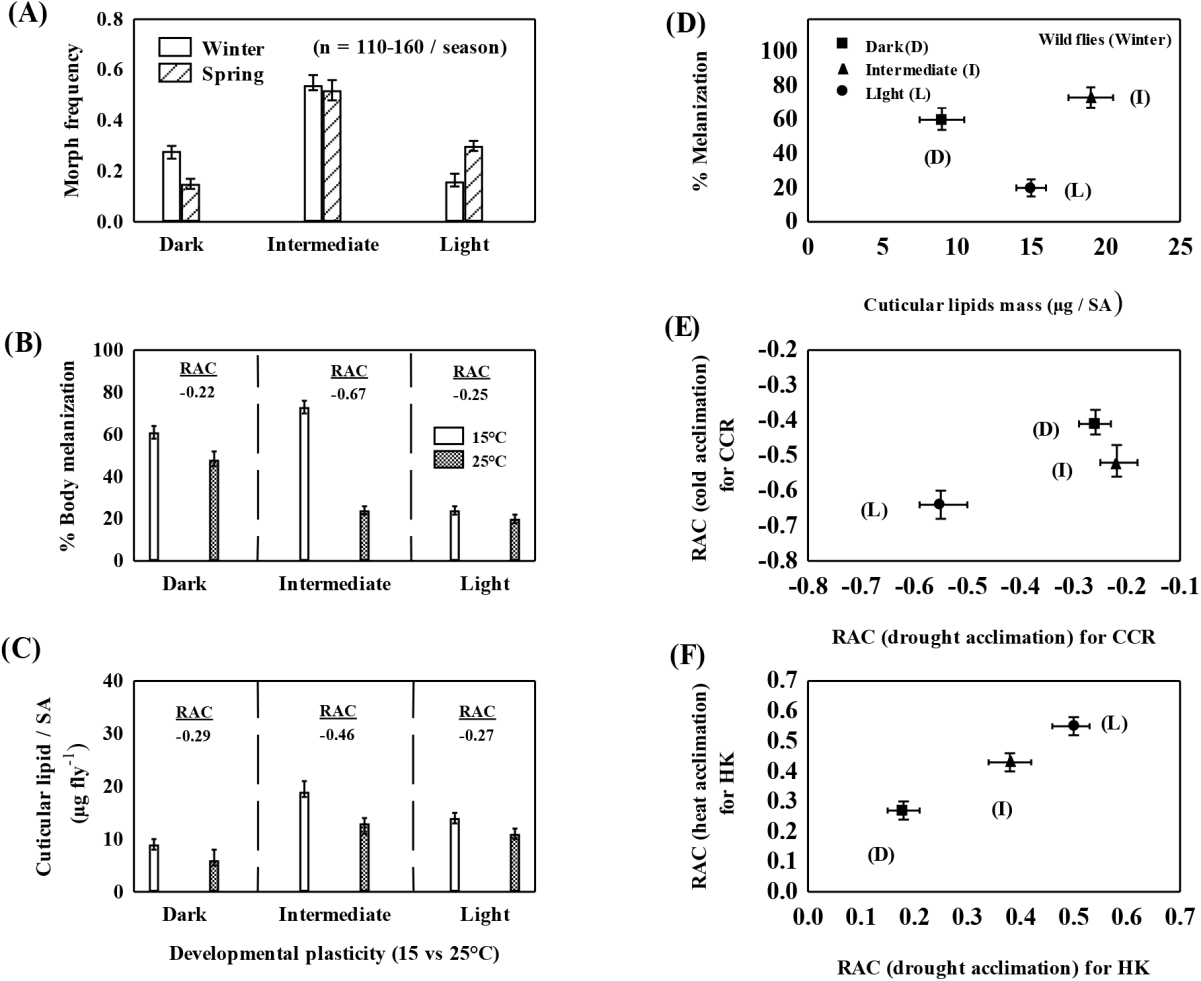
**(A) Seasonal (winter** versus **spring) changes in the mean frequencies of three body color morphs of *D. kikkawai* females collected during 3 years.** (B) Developmental acclimation (15 or 25°C) changes in percent body melanization, and (C) cuticular lipid mass/surface area (µg fly^−1^). RAC values are shown at the top of the bars (B,C). Body color morphs of winter collected flies differ in body melanization and cuticular lipid mass (D) and adult acclimation responses to cold or drought. (E,F) RAC based on (E) chill-coma recovery (CCR) after adult acclimation to cold or drought, and (F) heat knockdown (HK) after adult acclimation to heat or drought. For each trait, data (mean±s.e.m.) are based on three independent samples of ten wild-caught (winter) female flies of each body color morph.

Data (m±s.e.) on three body color morphs of *D. kikkawai* collected during winter [T_ave_; 14±2°C, 50±3% relative humidity (RH)] and spring (T_ave_; 24±2°C; 50±5% RH) of 3 years are shown in Fig. 1A and Fig. S1. The frequencies of body color morph flies varied across seasons, i.e. the frequency of light morph flies was 0.20±0.03 in winter and 0.30±0.04 during spring. In contrast, dark morph flies were more common during winter (0.30±0.04) as compared with spring (0.12±0.04). However, intermediate morph was abundant during both the seasons (0.44±0.06). For each morph and season, there were no significant differences in the frequencies across 3 years (Kruskal–Wallis test; *P*=0.36). Thus, local field collections revealed (a) occurrence of three body color morphs in *D. kikkawai* as compared with two morphs in other sympatric species of *montium* species subgroup, i.e. *Drosophila jambulina* and *Drosophila punjabiensis* (data not shown); (b) the ability of light morph to survive cold during winter; (c) greater abundance (∼0.45) of intermediate morph across winter as well as spring (Fig. 1A). Further, in Fig. 1B and C, we have shown changes in body melanization and cuticular lipid mass in response to rearing body color morph strains at 15 versus 25°C. For both the cuticular traits, intermediate morph showed greater plasticity while developmental plasticity effects were much reduced for dark or light morph strains (Fig. 1B,C). For wild-caught winter flies of *D. kikkawai*, we have shown morph specific changes in two cuticular traits (body melanization and cuticular lipid mass; Fig. 1D). Fig. 1 (E,F) shows significant differences in morph specific acclimation potential of winter collected flies. For cold versus drought acclimation, maximum response on CCR was observed in light morph as compared with the other two morphs (Fig. 1E). Acclimation capacity for heat versus drought was higher for light and intermediate morph as compared with dark morph (Fig. 1F).

### Cold or heat resistance of wild-collected body color morphs

Data (m±s.e.) on cold or heat resistance for basal level and after acclimation to cold or heat or low humidity conditions for independent samples of wild-caught flies of *D. kikkawai* (three body color morphs) are shown in Table 1. Chill-coma recovery time (CCR) of lighter morph flies of the control group was much faster as compared with flies of darker or intermediate morphs (Table 1). The relative acclimation capacity (RAC) due to cold acclimation was significantly higher for light morph as compared with dark morph. However, RAC values for rapid cold hardening were similar across morphs (Kruskal–Wallis test, *P*=0.26; Table 1). CCR of low humidity acclimated flies showed twofold higher acclimation capacity (RAC=−0.55) of light morph as compared with two other morphs (Table 1). Thus, in field captured flies, cold or humidity acclimation significantly increased cold tolerance of light morph despite its lower basal value of cold resistance (i.e. a trade-off between basal and acclimated capacity). The negative values of RAC for CCR indicate faster recovery and hence greater cold tolerance.

**Table 1.**
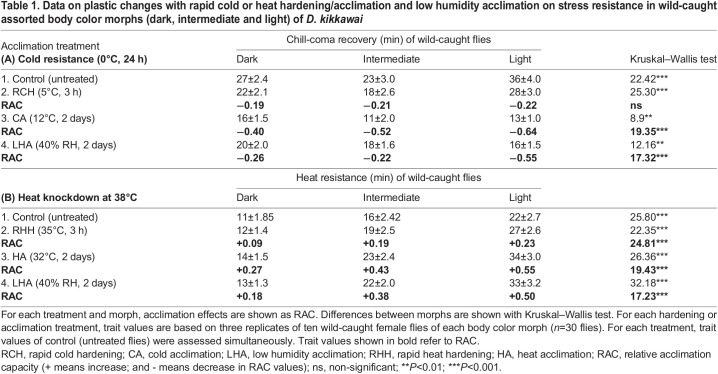
Data on plastic changes with rapid cold or heat hardening/acclimation and low humidity acclimation on stress resistance in wild-caught assorted body color morphs (dark, intermediate and light) of *D. kikkawai*

For heat resistance of wild-caught body color morph, data on RAC in response to rapid heat hardening or acclimation to heat or low humidity are given in Table 1. In winter collected flies, basal level of heat resistance was twofold higher in light morph as compared with dark morph (Table 1). Both heat hardening as well as heat acclimation; and after low humidity acclimation, light as well as intermediate morphs showed a significant increase in heat knockdown (high RAC values) as compared with dark morph (Table 1). Thus, hardening or acclimation significantly enhanced stress resistance of winter collected flies (Kruskal–Wallis test, *P*<0.01).

### Developmental plasticity changes due to low versus higher humidity or thermal conditions

Data on desiccation related traits, cold survival at 0°C and heat survival (at 38°C, 1 h) of three body color morph strains reared under different humidity conditions (40% versus 70% RH and at 25°C) are shown in Table 2. For each trait and body color morph, statistical differences across growth conditions are shown with Welch's test and ratio differences (Table 2). Low humidity (40% RH) rearing significantly increased cuticular lipid mass/fly, desiccation survival, and hydration level (Table 2). For body melanization and dehydration tolerance, changes due to low humidity were much reduced (∼10%) but cold survival at 0°C increased by ∼25% (Table 2). In contrast, rearing morph strains at 15°C significantly increased cold survival at 0°C (fivefold); desiccation resistance (twofold); and about 11–16% increase in dehydration tolerance but no effect on hydration level as compared with flies reared at 25°C (Table 2).

**Table 2.**
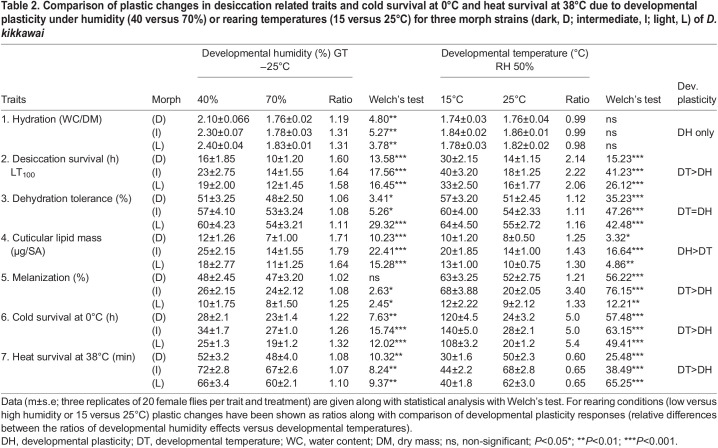
Comparison of plastic changes in desiccation related traits and cold survival at 0°C and heat survival at 38°C due to developmental plasticity under humidity (40 versus 70%) or rearing temperatures (15 versus 25°C) for three morph strains (dark, D; intermediate, I; light, L) of *D. kikkawai*

### Assessment of developmental plasticity changes

For different physiological traits, changes due to body color morphs and developmental plasticity and their interaction effects tested through ANOVA are shown in Table 3. Developmental plasticity effects (due to thermal rearing conditions) were about 70±5% for three traits (cold survival at 0°C, desiccation resistance and dehydration tolerance) while effects due to body color morphs were ∼17 to 27% (Table 3). In contrast, for three other traits (body melanization, cuticular lipid mass and heat survival) body color morphs contributed ∼60% of trait variance while developmental plasticity effects were about 30% (Table 3). Thus, developmental plasticity changes vary significantly for different physiological traits of *D. kikkawai*.

**Table 3.**
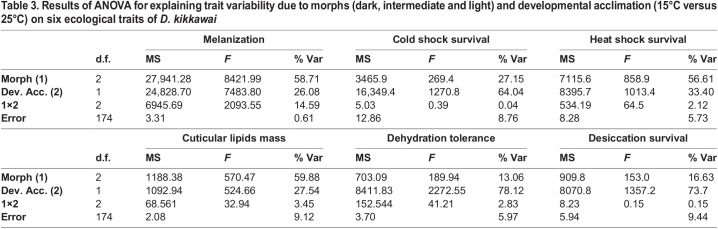
Results of ANOVA for explaining trait variability due to morphs (dark, intermediate and light) and developmental acclimation (15°C versus 25°C) on six ecological traits of *D. kikkawai*

### Plastic changes in cold tolerance due to cold or drought acclimation of adult flies

**Fig. 2.**
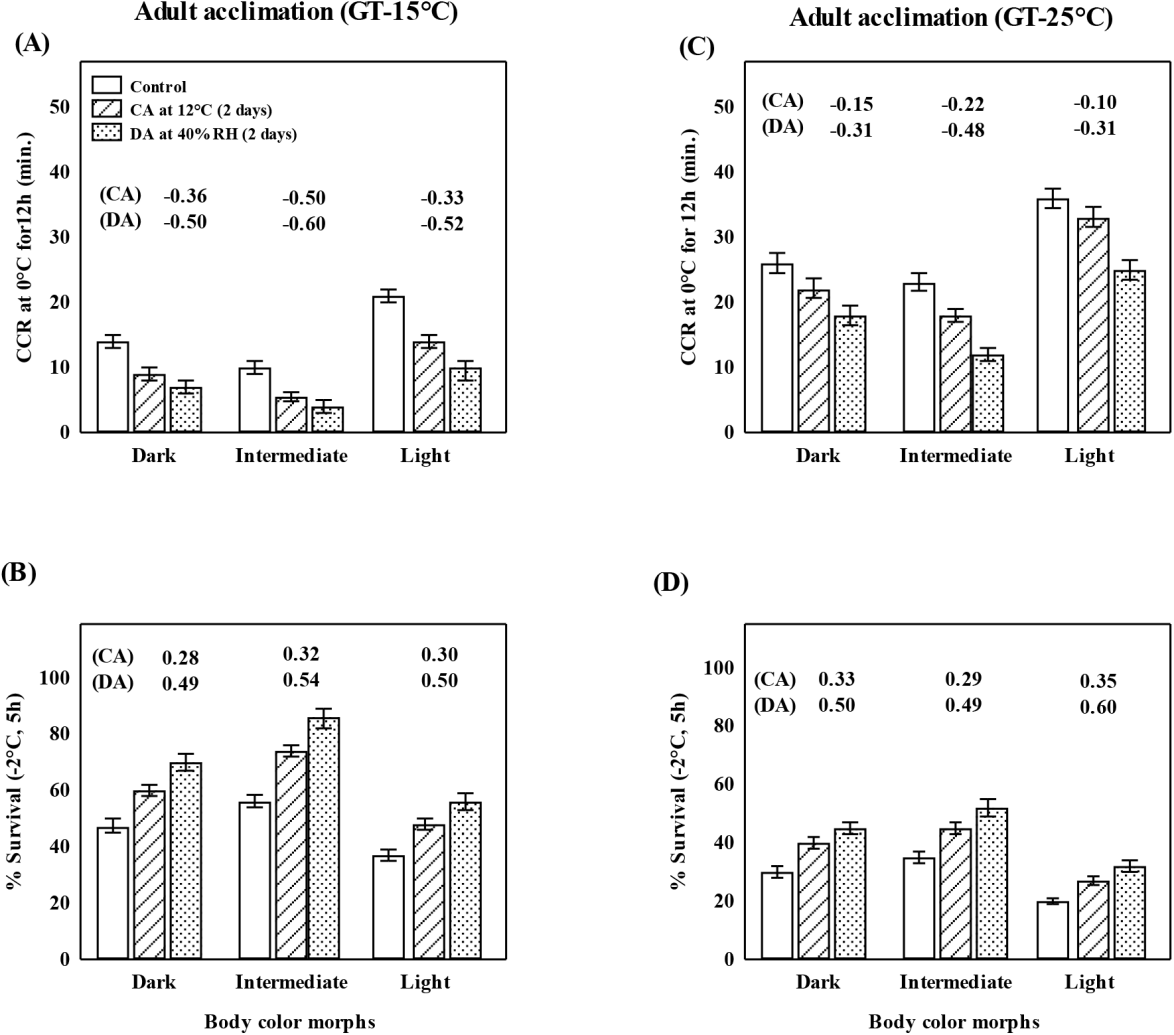
**Developmental and adult plasticity changes for cold tolerance (CCR: chill-coma recovery time; A,C); and percent cold shock survival at −2°C, 5 h (CSS; B,D) in three body color morphs of *D. kikkawai* reared at 15°C versus 25°C.** For adult acclimation, RAC values (shown on top of each bar) indicate greater cold tolerance after cold acclimation (CA: 12°C for 2 days) or drought acclimation (DA: 40% RH, 2 days). In A and C reduction in CCR denotes the increase in cold resistance. For each trait, data (m±s.e.) values are based on three replicates of 20 female flies of each body color morph. Statistical differences based on the Kruskal–Wallis test are significant (*P*<0.01). GT, growth temperature.

Plastic changes in cold resistance (chill-coma recovery time and cold shock survival) in three body color morph strains due to growth temperatures 15 versus 25°C and adult acclimation responses to cold (12°C, 2 days) or drought (40% RH, 2 days) are shown in Fig. 2 (A–D). For each morph and growth temperature (15 or 25°C), relative acclimation capacity values are shown on the top of bar diagrams. For light morph flies, cold shock survival was lower when reared at 25°C (Fig. 2). Flies reared at 25°C took two times longer duration of chill-coma recovery (min) than 15°C reared flies. For cold shock, basal survival values were higher for 15°C than 25°C (Fig. 2B,D). Basal values of chill-coma recovery and cold shock survival (Fig. 2) showed significant differences between morphs (Kruskal–Wallis test; *P*<0.01). For both the cold resistance traits, RAC values were higher for the intermediate morph as compared with the two other morphs. Further, an increase in cold tolerance was higher after drought acclimation as compared with cold acclimation (Fig. 2).

### Plastic changes in heat resistance due to heat or drought acclimation of adult flies

**Fig. 3.**
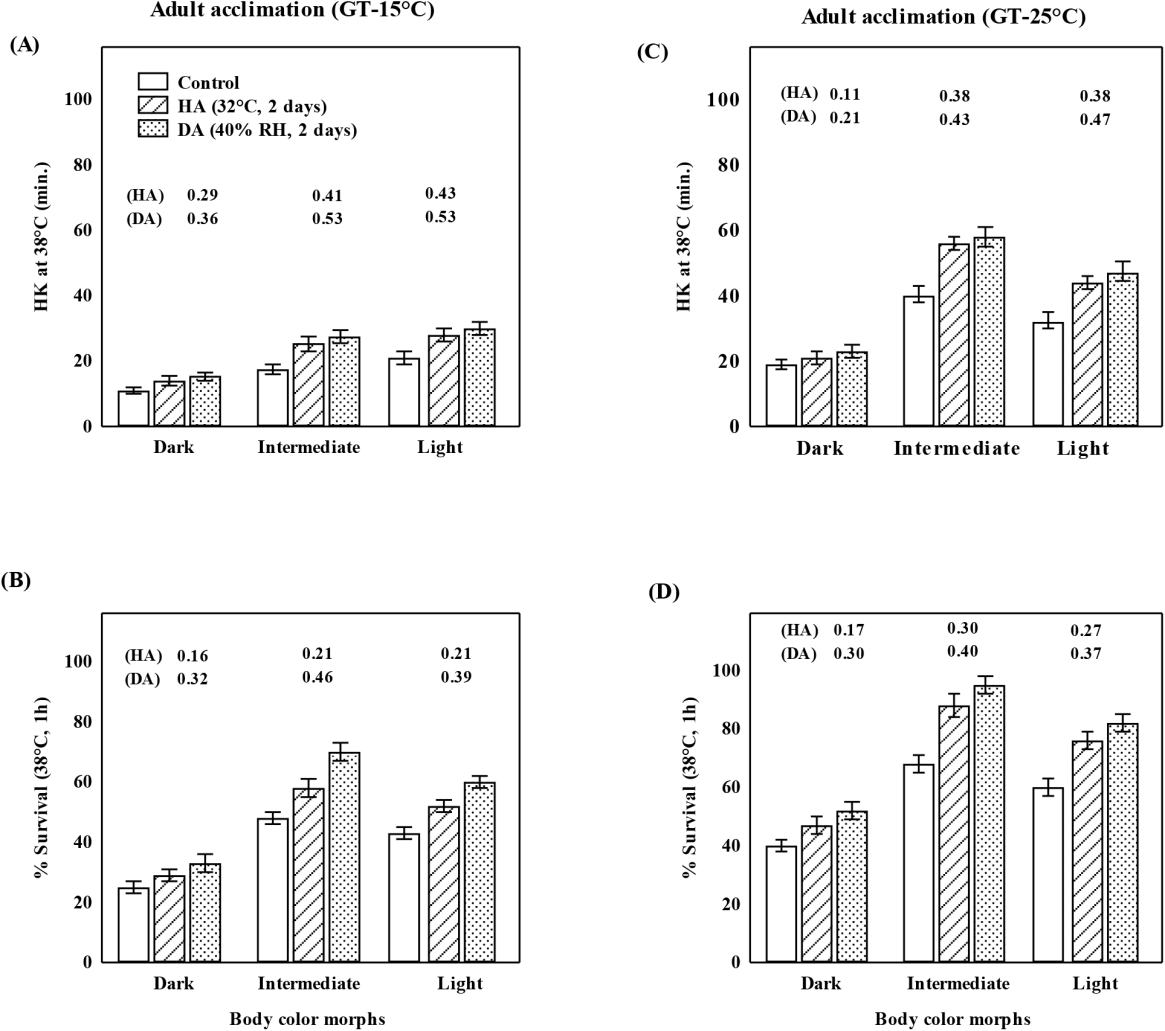
**Body color morphs of *D. kikkawai* show significant differences due to developmental plasticity (15 versus 25°C) for heat resistance (A,C: HK, and B,D: HSS at 38°C, 1 h).** Data (m+s.e.) for basal as well as after heat acclimation (HA: 32°C for 2 days) or drought acclimation (DA: 40% RH, 2 days) indicate greater heat tolerance of flies reared at 25°C than 15°C, intermediate as well as light morph is more resistant than dark morph flies. Adult acclimation responses are higher due to drought as compared with heat acclimation. For each morph, RAC (relative acclimation capacity) values are shown on top of each bar. For each trait, data (m±s.e.) values are based on three replicates of 20 female flies of each body color morph. Statistical differences between trait values of control and acclimated flies are significant (*P*<0.01) after Kruskal–Wallis test. GT, growth temperature.

Fig. 3 illustrates basal and acclimated levels of heat resistance (after heat or drought acclimation) of adult flies of three body color morph strains reared at 15 or 25°C. Heat resistance was measured with two metrics (Fig. 3A and C: heat knockdown time at 38°C; and B and D: heat shock survival at 38°C, 1 h). Lower heat resistance, as well as lower heat survival was evident in the dark morph. In contrast, heat resistance of intermediate morph was maximum with adult acclimation responses after heat or drought pretreatments (Fig. 3). A comparison of RAC reflects adult acclimation response to heat or drought pretreatments in the three body color morphs of *D. kikkawai* (Fig. 3). Drought acclimation-induced change in heat resistance was higher as compared with heat acclimation.

### Plastic effects of cold or drought acclimation on desiccation related traits

**Fig. 4.**
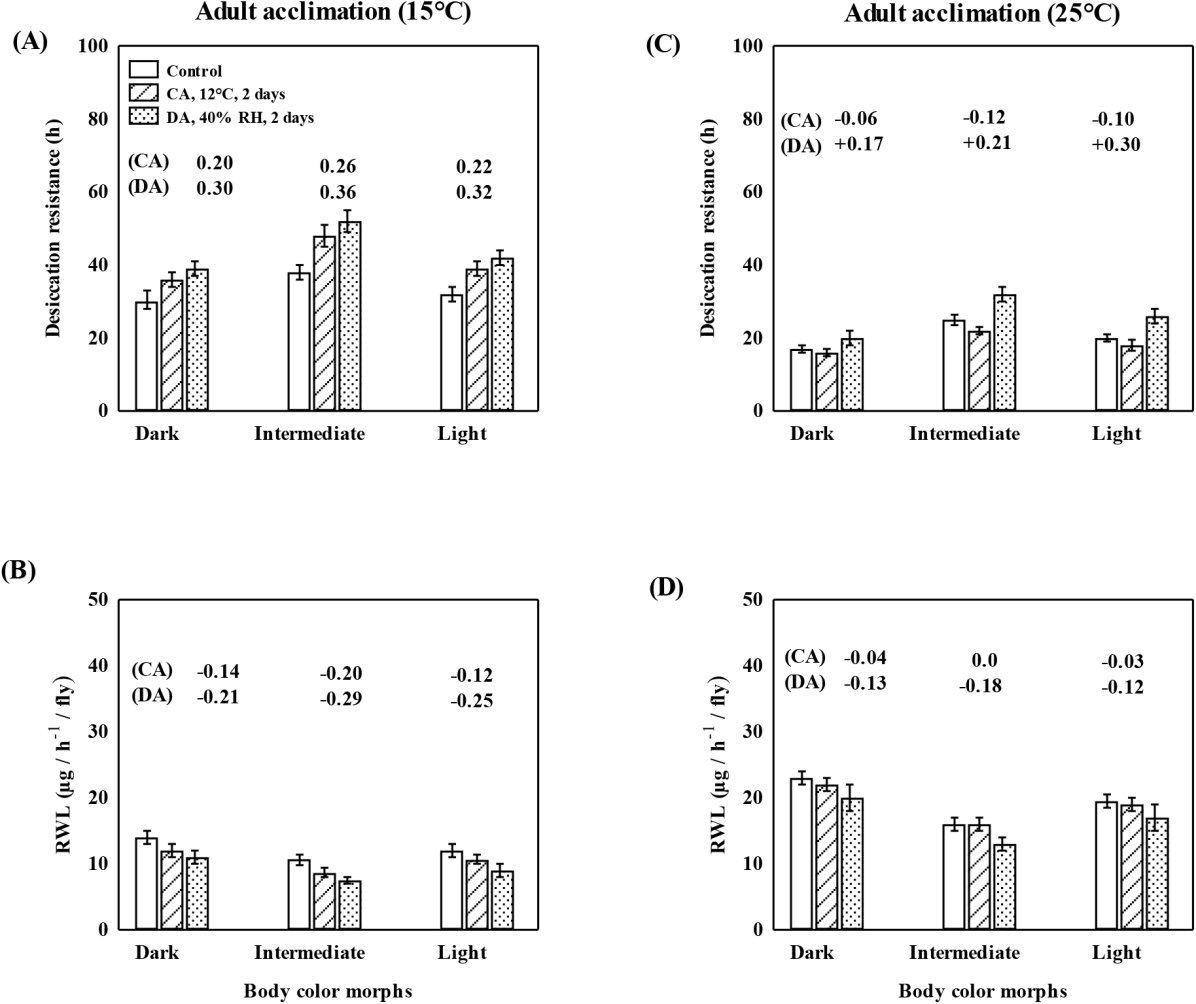
**Plastic changes in desiccation related traits in body color morphs (dark, intermediate and light) of *D. kikkawai*.** A and C show desiccation resistance, and B and D show rate of water loss (RWL) in response to developmental acclimation (15°C versus 25°C), and after adult acclimation (cold or drought). RAC are shown on top of bars and there are significant statistical differences between control and treatment groups (Kruskal–Wallis test, *P*<0.01). For each trait, data (m±s.e. values) are based on three replicates of ten female flies of each body color morph. GT, growth temperature.

In Fig. 4, bar diagrams represent basal as well as altered levels of desiccation resistance (A and C) or rate of water loss (B and D) as a consequence of adults acclimated to cold or drought. We found higher level of desiccation resistance in response to both cold and drought acclimation in morph strains reared at 15°C. Drought acclimation-induced changes were higher as compared with cold acclimation for flies reared at 15°C (Fig. 4). In contrast, for morph strains reared at 25°C, drought acclimation responses were significant but cold acclimation had a slightly negative effect on desiccation resistance as well as the rate of water loss (Fig. 4). Desiccation resistance of intermediate morph was higher at the basal level as well as after adult acclimation as compared to other two morphs reared either at 15 or 25°C. Similar responses were evident for the rate of water loss in the intermediate morph. For flies of dark morph reared at 25°C, desiccation resistance was lower along with a higher rate of water loss. Thus, we found morph specific differences in desiccation resistance across two growth temperatures. Finally, data on morph specific basal and acclimated levels of resistance to cold, heat and drought across two growth temperatures revealed significant differences (Kruskal–Wallis test, *P*<0.001).

### Impact of cold or heat hardening or acclimation on energy metabolites

In body color morph strains reared at 15°C, data on stress (cold or heat or low humidity)-induced changes in the levels of three energy metabolites (trehalose, proline, and body lipids) along with their relative acclimation capacity level (in bold) are shown in Table 4. The basal level of each energy metabolite was higher in light as well as intermediate morph as compared with dark morph (Table 4). For each morph, rapid cold hardening (RCH) increased the level of trehalose and proline (average RAC range=+0.25±0.3) but decreased the level of body lipids (average RAC range=−0.09). In contrast, rapid heat hardening (RHH) showed an opposite response, i.e. decrease in the level of trehalose and proline (average RAC range=−0.17) but increased the level of body lipids (average RAC range=+0.10, Table 4). Thus, hardening-induced changes were different under cold or heat in all three morphs (Table 4). In contrast, we found shared patterns of accumulation of trehalose but utilization of proline under acclimation to cold or heat. Further, low humidity acclimation followed by either rapid cold hardening or rapid heat hardening exhibited utilization of trehalose but accumulation of proline (Table 4). These observations support complementary changes induced by low humidity acclimation versus cold or heat acclimation. Further, acclimation changes in body lipids showed independent patterns under cold or heat acclimation and with humidity acclimation. For each morph, cold acclimation increased but heat acclimation decreased the level of body lipids. In contrast, low humidity with rapid heat hardening increased the level of body lipids but a significant decrease under the combined effect of RCH and low humidity (Table 4). Therefore, we found divergent patterns of hardening versus acclimation changes in total body lipids. Finally, both in terms of the amount of each energy metabolite as well as energy budget (calculated with standard conversion factors), our data favored complementary changes for cold versus drought (or heat versus drought) for possible maintenance of energy balance.

**Table 4.**
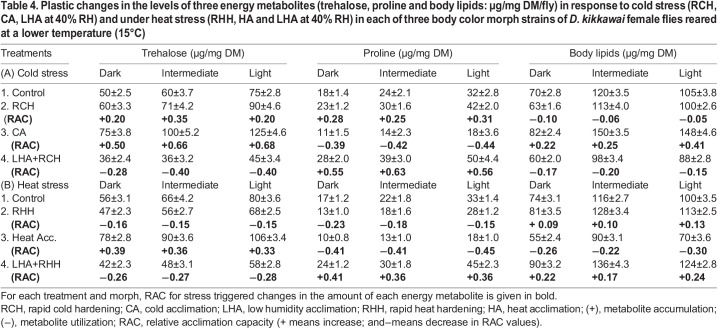
Plastic changes in the levels of three energy metabolites (trehalose, proline and body lipids: µg/mg DM/fly) in response to cold stress (RCH, CA, LHA at 40% RH) and under heat stress (RHH, HA and LHA at 40% RH) in each of three body color morph strains of *D. kikkawai* female flies reared at a lower temperature (15°C)

## DISCUSSION

Adaptations to colder and drier climatic conditions are a major challenge for tropical *Drosophila* species. Adult acclimation of body color morphs of *D. kikkawai* revealed enhanced levels of cold resistance. We found supporting evidence for plastic changes in the level of stress resistance and energy metabolites on the basis of development plasticity (rearing flies at 15°C), followed by adult acclimation to cold, drought or heat. We observed a trade-off between basal and acclimated levels of resistance to cold or heat or drought in the three body color morph strains. Thus, plastic changes in the cold or drought tolerance of *D. kikkawai* seem consistent with the ability of this tropical species to invade other territories. However, it may be mentioned that *D. kikkawai* flies have not been recorded from high latitudinal temperate regions and there are no data on overwintering of this species in extremely cold environments (Bock, 1980; Ashburber et al., 2003; Markow and O'Grady, 2006).

### Adult acclimation potential of winter collected wild flies

In *Drosophila*, several studies have reported an increase in cold or heat resistance in response to cold or heat hardening/acclimation (Hoffmann et al., 2003; Angilletta, 2009; Overgaard et al., 2011; Nyamukondiwa et al., 2011). In *D. melanogaster*, cold acclimation increased cold tolerance but at a cost of a decrease in heat resistance (Kristensen et al., 2008). In contrast, during winter field acclimatized flies of *Drosophila subobscura* from Denmark revealed a lack of trade-off between resistance to cold or heat (Sørensen et al., 2015). Thus, there is a need to simultaneously analyze thermo-resistance of field captured as well as laboratory-reared flies of different tropical *Drosophila* species (Hoffmann, 2010). In wild-caught flies of light morph, low humidity acclimation significantly increased resistance to cold (RAC=−0.55 for CCR) and for heat (RAC=+0.50) while such effects were lower for the dark morph. The cold acclimation capacity of field collected light morph flies is significantly higher (RAC=−0.64). Both basal and acclimated levels of heat resistance are also higher in the case of light as well as intermediate body color morphs. A lack of trade-off between cold or heat resistance in the light morph and intermediate morph is likely to support the invasive potential of *D. kikkawai*. The present results on *D. kikkawai* are consistent with a lack of trade-off between cold and heat resistance in *D. subobscura* (Sørensen et al., 2015). Further, in *D. kikkawai*, the persistence of lighter body color morph under cooler (10 to 15°C) and drier (40 to 50% RH) environments is not consistent with the thermal-melanism hypothesis (Majerus, 1998; True, 2003). According to this hypothesis, darker morph is able to absorb solar radiations as compared with lighter body color morph of diverse insect taxa living under colder environments (Brakefield and Willmer, 1985; True, 2003). For the abundance of light body color morph of *D. kikkawai* during winter, the possible explanations include greater cold acclimation capacity; and more amount of cuticular lipids to cope with drier conditions associated with colder environments (10–15°C). We also observed higher storage level of proline, trehalose, and total body lipids in the light body color morph as compared other two morphs.

### Cold or drought-induced plasticity changes in cold tolerance

Insects from temperate regions are able to improve their survival under cold conditions as a consequence of prior exposure to RCH or cold acclimation (Overgaard et al., 2007; Kostal et al., 2011; Colinet et al., 2012). In contrast, tropical insects are stenothermal (limited range of developmental temperatures) and their cold sensitivity is associated with restricted distribution in the tropics (Kellermann et al., 2009; Hoffmann, 2010). In two tropical *Drosophila* species, *D. malerkotliana* and *D. suzukii*, developmental plasticity and adult acclimation responses are able to increase tolerance to cold (Parkash et al., 2013; Shearer et al., 2016). Such analysis has received less attention for other tropical *Drosophila* species. In the present study, *D. kikkawai* morph flies (reared at 15°C) could survive for 5 to 6 days at 0°C but 25°C flies survived only for 1 day. There are morph specific differences in the basal level of cold resistance assessed through chill-coma recovery and cold shock survival, i.e. basal cold tolerance of intermediate morph, as well as dark morph, was higher than light morph. However, adult acclimation response with desiccation acclimation (40% RH, 2 days) significantly increased cold resistance (∼60%) as compared to cold acclimation (∼30–40%); and acclimation changes were higher for the intermediate morph. In contrast, for *D. kikkawai* morphs reared at 25°C, both basal levels as well as cold acclimation responses were lower but drought acclimation effects were higher. This reflects higher protective effects of drought acclimation as compared with cold acclimation for body color morph strains of *D. kikkawai* reared under lower or higher thermal conditions. It may be argued that simultaneous assessment of cold or drought acclimation for plastic changes in cold tolerance are important for assays involving cold-induced mortality in different insect taxa from the tropics.

### Plasticity changes in the drought resistance

Water conservation is a major challenge for survival under colder or drier conditions (Chapman, 1998). Three physiological mechanisms help to conserve body water, i.e. extra water storage (or hydration level); reduction in the rate of water loss through the cuticle; and dehydration tolerance (Hadley, 1994; Gibbs et al., 1998, 2003; Kalra et al., 2014). Comparative studies on *Drosophila* species (reared at 25°C and tested under <10% RH) have shown increase in the level of the desiccation resistance in response to drought hardening both for generalist as well as tropical *Drosophila* species (Hoffmann, 1991; Kellermann et al., 2018). However, these previous studies did not consider developmental acclimation-induced changes in physiological mechanisms of water conservation. In the present work, we compared plastic changes in body melanization and cuticular lipid mass, hydration level, desiccation resistance, dehydration tolerance due to different developmental humidity conditions (low versus high humidity) for morph strains reared at 25°C (as a proxy of spring season). The developmental plasticity due to low humidity (40% RH) resulted in significant levels of increase in the water storage or hydration level (∼20–30%), cuticular lipid mass (∼60–80%), desiccation resistance (∼60%) while plastic changes in the dehydration tolerance were lower (<10%). Thus, developmental plasticity due to low humidity could increase drought related physiological traits consistent with adaptations to warmer and drier ambient conditions in the tropics.

To understand developmental plastic changes induced by rearing temperatures, *D. kikkawai* morph strains were reared at 15°C versus 25°C. Developmental plasticity effects were morph dependent, i.e. lower growth temperature-induced plastic effects (threefold higher body melanization; 1.5-fold higher cuticular lipid mass; and twofold desiccation resistance) and such plastic changes were higher for intermediate body color morph as compared with dark and light morphs. There were no plastic changes in the water storage or hydration level; and only a modest increase in dehydration tolerance (∼11–16%) as a consequence of rearing morph strains at 15°C. Therefore, body color morphs of *D. kikkawai* are able to significantly enhance desiccation tolerance through rearing at cooler temperature (15°C). Further, both cold and drought acclimation (of adult flies of all the three body color morphs of *D. kikkawai*) significantly increased (∼25%) desiccation resistance; and reduction in the rate of water loss (∼12–20%). In *D. kikkawai*, plastic changes due to developmental and adult acclimation resulted in morph specific physiological differences for coping colder and drier environmental conditions.

### Relationship between basal and induced stress resistance

Developmental and/or adult plastic responses are quantified in terms of RAC, which helps in comparing the benefits or cost of plasticity effects across *Drosophila* species and populations (Kellett et al., 2005; Angilletta, 2009; Sgrò et al., 2010). Rearing temperatures are known to alter the levels of acclimation capacity in *D. melanogaster*, i.e. RAC value of 0.30 for flies reared at 18 or 25°C (Cavicchi et al., 1995). We also found no effect of rearing temperatures (15 or 25°C) on the relative acclimation capacity of all the three morphs of *D. kikkawai* for cold tolerance. In contrast, for adult acclimation responses, we found morph-specific differences in the level of cold resistance. Average relative acclimation capacity based on cold shock survival was similar (∼ 0.30) for cold acclimation but significantly higher due to drought acclimation (RAC ∼0.50). For heat resistance of body color morphs, drought acclimation capacity was significantly higher than heat acclimation capacity, and plastic responses were similar for the intermediate as well as light morphs. Thus, the ability to increase stress resistance through hardening/acclimation to different climatic stressors (cold, heat or drought) seems consistent with ecological success of invasive *D. kikkawai*.

### Plastic changes in the energy metabolites

In insects, fat body (adipocyte cells) synthesizes carbohydrates, lipids, proteins and amino acids and serves as a central depot for energy metabolites (Chapman, 1998). In response to energy demands (for flight, reproduction, growth, etc.), fat body cells release energy metabolites to body cells via hemolymph (Arrese and Soulages, 2010). For insect flight, energy demands are provided by circulating energy metabolites, i.e. proline, trehalose and lipids (Bursell, 1981). Therefore, we assumed that these three energy metabolites are likely to serve as preferred resources under stress-induced plastic changes in *Drosophila* species. We examined such an expectation in a previous study on *Drosophila immigrans* (Tamang et al., 2017). We found cold- or drought-induced complementary plastic changes in the accumulation and utilization of proline and trehalose (Tamang et al., 2017). In three body color morph strains of *D. kikkawai*, we extended that work by investigating plastic changes in three energy metabolites in response to cold or heat versus drought hardening or acclimation.

In *D. melanogaster*, cold-induced [rapid cold hardening (RCH) or cold acclimation (CA)] changes in energy metabolites have received greater attention (Overgaard et al., 2007; Kostal et al., 2011; Colinet et al., 2012). However, previous studies on *D. melanogaster* did not consider drought- as well as cold-induced changes in energy metabolites simultaneously. In contrast in the cold-hardy *Belgica antarctica*, metabolic changes induced by heat shock (30°C, 1 h) or freezing (−10°C, 6 h) or desiccation (4°C, 98.5% RH) revealed upregulation of some energy metabolites due to freezing or desiccation while heat or desiccation downregulated other metabolites (Michaud et al., 2008). In another study on *B. antarctica*, slower dehydration-induced increase in the trehalose level, which increased survival to cold or heat stress (Benoit et al., 2009). These studies on *B. antarctica* have revealed protective effects of drought on cold or heat resistance as well as in the regulation of energy metabolites for adaptations to harsh climatic conditions. In contrast to the freeze tolerant *B. antarctica*, cold-induced plastic changes in the chill-susceptible *D. melanogaster* increased the level of trehalose but not for the proline content (Overgaard et al., 2007). In two subtropical species reared at 21°C, increased level of desiccation resistance in *D. kikkawai* is associated with higher storage of carbohydrates as compared with its sibling *D. leontia* (Ramniwas and Kajla, 2012). In this study, plastic changes in the levels of energy metabolites (carbohydrates, body lipids, and proteins) were not investigated in response to drought acclimation (Ramniwas and Kajla, 2012). However, in the present work on *D. kikkawai*, we tested whether cold versus drought, or heat- versus drought-induced plastic changes in three energy metabolites result in energy balance or not.

In *D. kikkawai*, the plastic changes in energy metabolites induced by RCH or RHH showed opposite patterns, i.e. RCH increased the level of trehalose and proline but decreased lipids. In contrast, RHH decreased trehalose and proline but increased lipids. Further, we found that plastic changes in three energy metabolites are stressor dependent (i.e. thermal versus humidity acclimation) and result in shared patterns for trehalose and proline but different for body lipids. For example, cold or heat acclimation led to trehalose accumulation but utilization of proline. In contrast, low humidity acclimation showed the opposite pattern, i.e. utilization of trehalose but the accumulation of proline. Thus, cold or heat versus drought acclimation resulted in complementary changes to maintain energy balance. For plastic changes in proline, a possible explanation is that reactive oxidative stress (ROS) caused by either cold or heat stress could be quenched through proline utilization (Michaud et al., 2008). In contrast trehalose – being a chaperone molecule – accumulation under cold or heat could confer stability to cellular macromolecules (membranes, proteins, etc.) to reduce the detrimental effects of thermal stress (Overgaard et al., 2007). Also, trehalose serves as an osmolyte to give protection against desiccation stress (Benoit et al., 2009). In *D. kikkawai*, cold or heat acclimation-induced changes in body lipids (accumulation under cold but utilization under heat) revealed different patterns when compared with trehalose or proline. Despite morph-specific differences in the level of acclimation effects, cold and drought (or heat and drought)-induced changes have shown buffering effects for maintaining energy balance. Finally, in terms of energy budget (based on three metabolites only), intermediate morph and light morph (6.3 J/mg) showed about 20% higher energy budget as compared with dark morph (5.2 J/mg). For each morph and energy metabolite, changes in the energy budget due to effects of RCH or RHH and acclimation (CA or HA or LHA) indicate a buffering effect for homeostasis level.

### Impact of climate and urbanization

*Drosophila kikkawai* is one of the few tropical *Drosophila* species that has invaded other continents and attained the status of subcosmopolitan species (Pinheiro and Valente, 1993). The present work on *D. kikkawai* from its native range supports the ability of this species to increase tolerance to cold or drought through plastic changes (developmental and adult acclimation at 15°C). However, the levels of plasticity of cold or drought resistance may differ between *D. kikkawai* populations from its native range and those from invaded territories on other continents. Further, the body color morphs of *D. kikkawai* are likely to differ in their behavioral adaptations to colder and drier environments, and to hot and drier conditions. For example, the percent distribution of darker females of *D. kikkawai* collected from three urbanization zones (in the Brazilian city of Porto Alegre) revealed significant differences, i.e. higher frequency of darker morph (60%) in low urbanization zones (with relatively lower thermal conditions) as compared to 40% in the warmer zones due to higher urbanization of the same city and during the same season (Pinheiro et al., 2003). Therefore, urbanization can influence abundance levels of body color morph of *D. kikkawai*. The present study did not consider the physiological basis of behavioral divergence of different body color morphs of *D. kikkawai* across urbanization zones, which also vary in thermal and drought stress levels. However, the impact of climate and urbanization was addressed in the case of invasive subspecies *Aedes aegypti aegypti* from sub-Saharan African localities (Rose et al., 2020). Rose et al. demonstrated that 65% of behavioral trait variability (human biting) was associated with dry season intensity, while ∼18% variation in human biting behavior was accounted due to urbanization (Rose et al., 2020). In *A. aegypti aegypti* populations collected from twenty-seven localities of sub-Saharan Africa, a significant correlation was found between variable levels of darker versus lighter abdominal scales and seasonal aridity index changes across localities. Thus, there is a need to explore the impact of urbanization on the abundance levels of body color morphs of *D. kikkawai*.

## MATERIALS AND METHODS

### Collections and cultures

We collected wild *Drosophila* flies with the help of net sweeping and through bait-traps from Rohtak (Lat: 28.89°N; Alt: 220m) during winter (December of 2015 to 2017) and spring (March of 2016 to 2018). Out of wild-collected flies (*n*=∼500-600), *D. kikkawai* flies were identified following Parshad and Paika, (1964). The mean frequency of each body color morph was calculated as a number of females of each morph divided by total number of *D. kikkawai* female morphs collected during each season. True breeding strains for all the three morphs were isolated and maintained at 20°C. Male flies of *D. kikkawai* lack melanization and were not investigated in the present study. We reared laboratory cultures at low density in culture bottles (250 ml) on a standard food medium (comprising maize powder, yeast powder, agar-agar, jiggery, and water). For each body color morph strain, flies laid eggs at 20°C, 60% RH for 6 h in each of ten bottles. Five culture bottles with eggs were kept in one of two different Metrex environmental chambers (www.metrexscientific.in), which provided humidity (25°C and 40% RH) or (25°C and 70% RH) for egg to adult development. Likewise, for each morph strain, eggs laid at 20°C in food bottles were transferred either at 15 or 25°C with 50% RH in separate incubators. *Drosophila kikkawai* flies reared under low humidity conditions did not show adverse effects on the egg to adult viability (∼83±4% for morphs) and this may be likely due to species desiccation resistance or adaptability to drier seasons under natural habitats (Parkash and Ranga, 2013). Further, *D. kikkawai* morph flies were held under rearing conditions throughout development from egg to adult stages; and post eclosion virgin flies were kept in food vials at the given temperature. In the present work, we used virgin female flies (6 days old) of each of three body color morph strains of *D. kikkawai*. Therefore, the reproductive status of virgin female flies was less likely to affect stress resistance traits related to heat, cold or drought. We used experimental cultures, i.e. egg-laying restricted to 6 h, removal of parental flies from culture bottles, followed by the development of flies under constant thermal conditions, isolating batches of virgin flies and ageing these flies in separate food vials and analyzing 6-day-old flies

### Establishment of true breeding strains for dark, intermediate or light body color morphs

Mass culture of *D. kikkawai* with field-collected 30 males as well as 30 females was maintained in the laboratory at 20°C for five generations on a standard food medium. For true breeding strains for each of three body color morphs of *D. kikkawai*, progeny of independent single pair matings (*n*=80) were established followed by checking the progeny of each pair. Out of these vials, single pair matings showing segregation patterns of dark or light females were not considered further. However, single pair matings with only darker female progeny up to five generations were considered as dark body strain. Similarly light body color strain was identified. Each of these strains laid eggs at 20°C and two food bottles (250 ml) with eggs were transferred to each of two growth temperatures (15 or 25°C). As stated above, body color of progeny of each strain was checked across both temperatures. Dark or light strains, which showed reduced level of plasticity across 15 or 25°C, were treated as true breeding dark or light strains. However, some dark strains revealed higher level of plasticity, i.e. darker at 15°C but lighter at 25°C and such strains were treated as true breeding intermediate strains. Finally, four strains were pooled to establish a true breeding dark strain of *D. kikkawai*. This approach was followed in case of light strain mass culture as well as for intermediate body color morph strain. We maintained each morph strain independently for three generations before doing the experiments.

### Experimental set-up

In winter collected morph specific samples (three replicates of ten wild-caught flie/morph) of *D. kikkawai*, we assessed basal level and plastic changes in cold resistance due to adult hardening or acclimation responses. Cold resistance was measured on the basis of chill-coma recovery (after keeping fly groups at 0°C for 24 h) in four sets of control and treatment groups (a) rapid cold hardening at 5°C for 3 h; (b) cold acclimation at 12°C for 2 days; (c) low humidity acclimation at 40% RH for 2 days. For heat resistance, we measured plastic changes in heat knockdown at 38°C in control and treatment groups of flies (a) rapid heat hardening at 35°C, 3 h; (b) heat acclimation at 32°C for 2 days; (c) low humidity acclimation (40% RH, 2 days). Plastic changes due to adult acclimation (hardening or acclimation to cold or heat or low humidity were assessed in terms of RAC in order to avoid possible effects due to independent samples.

We did pilot experiments for chill-coma recovery and heat knockdown on independent random samples of winter collected female flies of each morph. There was variation in trait values between samples probably due to age or nutrition under field conditions. Thereafter, we took body weight of each wild-caught fly and grouped flies for each morph specific sample (with a wet weight variation of ∼10%). Independent samples based on this criteria reduced variation between samples for trait values (chill-coma recovery or heat knockdown). For assessment of stress resistance (cold or heat), winter collected fly samples were kept in the laboratory at 18°C for 24 h to acclimatize flies. For each trait and morph, basal trait values and after hardening or acclimation were assessed in three independent samples.

### Assessment of developmental plasticity effects in body color morph strains

Different sets of experiments were conducted with laboratory reared body color morph strains. For each experiment, three replicates of 20 virgin female flies (6 days old) were used. First, developmental acclimation effects of low versus high humidity (40 versus 70% RH and growth temperature 25°C) were assessed for physiological traits related to desiccation resistance; and for cold or heat survival so as to find effects of wet or dry conditions (as a proxy of spring season). For these stress related traits, effects of rearing thermal conditions (15 or 25°C and 50% RH) were also conducted. Second, in laboratory reared flies (15 or 25°C) of body color morph strains, we analyzed changes in basal trait values (resistance to cold, heat or drought) in response to developmental plasticity. For adult acclimation, three different experiments (cold or heat acclimation versus low humidity acclimation) were carried out for stress related traits. Controls flies were not acclimated (untreated). Third, for each treatment experiment, separate controls were run simultaneously. Possible changes due to age effects were avoided by controlling the age of adult flies (6-day-old virgin flies) for control versus treatment experiments (see, Fairbanks and Burch, 1970). Fourth, for each morph strain, changes in the level of three energy metabolites (trehalose, proline or body lipids) were analyzed in control (untreated), and flies subjected to hardening/acclimation pretreatment related to either cold versus drought, or heat versus drought.

### Analysis of body melanization and cuticular lipid mass in body color morph strains

Developmental plastic changes in body melanization and cuticular lipid mass were assessed in morph specific fly groups (three replicates of 20 flies/morph strain) reared under different conditions (15 versus 25°C, or 40 versus 70% RH). Among the female flies of *D. kikkawai*, identification of dark and light morphs was easier but intermediate flies were identified based on melanization patterns of second, third and fourth abdominal segments (Fig. S1). In flies of intermediate morph reared at 15°C, fifth, sixth and seventh abdominal segments were fully melanized but there was no melanization in these segments in flies reared at 25°C. However, dark and light morph flies revealed ∼25% reduced level in overall melanization when reared at 25°C as compared to 15°C. Abdominal melanization was estimated from the dorsal view of the female abdomen giving values ranging from 0 (no melanization) to 10 (complete melanization) for each of the six abdominal segments (second to seventh). Since the abdominal segments differ in size (i.e. 0.86, 0.94, 1.0, 0.88, 0.67 and 0.38 for second to seventh segments, respectively), these relative sizes were multiplied with segment-wise melanization scores. Data on percent melanization were calculated as (Σ observed weighted melanization scores of abdominal segments per fly/Σ relative size of each abdominal segment×10 per fly)×100 (Parkash et al., 2008). Further, for estimation of cuticular lipid mass, 6-day-old flies were used (three replicates of 20 flies of each body color morph). Individual flies were dried overnight at 60°C to obtain dry mass, i.e. devoid of body water. Each dried fly was kept in HPLC-grade hexane for 5 min and again dried at room temperature and finally reweighed on a Sartorius microbalance (model CPA26P, with precision 0.001 mg; www.sartorius.com). Cuticular lipid mass per fly was calculated as the difference in mass following hexane extraction divided by surface area (µg/SA).

### Analysis of developmental or adult plasticity changes in desiccation related traits

Desiccation survival was measured as LT_50_ and LT_100_ in dry air (<10% RH) for three replicates of 20 flies of each body color morph. To estimate total body water content, rate of water loss; and dehydration tolerance, individual flies were used. For each morph strain, individual flies were weighed on the Sartorius microbalance (model CPA26P; 0.001 mg precision) and then reweighed after drying for 24 h at 60°C. For the calculation of the rate of water loss, we followed Wharton's method (Wharton, 1985). Total body-water content (*m*) was calculated as the difference between wet or fresh (*f*) and dry (*d*) masses, i.e. *m*=*f*−*d*. Individual flies were weighed and exposed to approximately 10% relative humidity for a specified time at 1 h intervals (1–8 h), and then reweighed. The rate of water loss was derived from the slope of regression line on a plot of ln (*mt*/*m*_0_) against time according to Wharton’s exponential equation: *m_t_=m*_0_e^-^*kt*, where *m_t_* is the water mass at time *t* and *m*_0_ is the initial water content (Wharton, 1985). Rate (*kt*) is the slope of the regression line. Further, to estimate dehydration tolerance (the ability to tolerate water loss at death under desiccation stress), we measured water content of individual flies close to the time of death (under 10% RH), and the water content remaining was also determined (Gibbs et al., 1997). The dehydration tolerance was estimated as the percentage of total body water lost until death due to desiccation. Dehydration tolerance was calculated as: [(wet body mass−body mass at death)/(wet body mass−dry body mass)×100].

### Analysis of cold tolerance in laboratory reared flies

We used four sets of experiments to measure cold resistance, i.e. (a) cold survival at 0°C as a function of different time durations and LT_50_ and LT_100_ were calculated from the survival curves, (b) chill-coma recovery experiments, (c) cold shock survival, (d) rapid cold hardening or cold acclimation. Flies of each morph (three replicates of 20 flies) were transferred without anesthesia into empty 10 ml glass vials placed in thermocool boxes containing ice flakes. This setup was kept at 0°C in the refrigerator. The vials were removed after 12 h followed by the transfer of flies to Petri plates (9 cm diameter) in a temperature-controlled room at 21°C and the chill-coma recovery period (in minutes) was recorded. The flies were considered recovered when they were able to stand on their legs. For cold acclimation, adult flies were pretreated to 12°C for 2 days and allowed to recover at 15°C for 6 h before assessing chill coma recovery (Rako and Hoffmann, 2006).

For cold survival, adult females (three replicates of ten flies for each cold treatment duration) were subjected to 0°C for different durations, 5, 10, 15, 20, 25, 30, 35, 40 h for 25°C reared flies, and 20, 40, 60, 80, 100, 120, 140, 160 h for flies reared at 15°C. Further, for cold shock mortality, adult flies (of each body color morph) were subjected to −2°C for 5 h followed by returning flies at their respective growth temperatures (15 or 25°C) and observing mortality after every 24 h up to 3 days. However, the data were represented as percent survival.

### Analysis of heat resistance in the control and acclimated groups of flies

Different sets of experiments for simultaneous analysis of control and treatment groups of flies of each morph strain were performed to assess (a) effect of rearing conditions (40 versus 70% RH, or 15 versus 25°C) on heat survival duration at 38°C, (b) heat knockdown time in adult flies of control and acclimated groups (heat or drought), and (c) percent survival after heat shock at 38°C for 1 h in control and acclimated fly group. For heat knockdown, individual adult females of each morph of *D. kikkawai* (three replicates of 20 flies) were placed in 5 ml glass vials submerged in a water bath set at a constant temperature of 38°C. Heat resistance was scored as the time taken for flies to be knocked down. Flies were heat acclimated for 2 days at 32°C followed by 6 h recovery at respective growth temperature before assessing heat knockdown resistance. For analysis of rearing conditions (temperatures or humidity) effects, heat stress mortality at 38°C was assessed in fly groups subjected for 10, 20, 30, 40, 50, 60, 70 and 80 min independently and LT_50_ and LT_100_ values were calculated from the survival curves. We observed percent mortality after heat shock (at 38°C, 1 h) in control and heat or drought acclimated flies. However, the data were represented as percent survival.

### Estimation of plastic changes in energy metabolites

Different sets of experiments were performed to analyze plastic changes in three energy metabolites (trehalose, proline, and total body lipids) in control (untreated) and morph specific fly groups subjected to rapid hardening or acclimation treatments.

### Body lipids estimation

Body lipid content was estimated in individual flies (three replicates of ten flies of control as well as hardened or acclimated) of three body color morph strains. Individual flies were dried at 60°C for 48 h, and dry mass was obtained by weighing on a Sartorius microbalance (model CPA26P; 0.001 mg precision). Body lipids were extracted by placing individual flies in 2 ml centrifuge tubes (http://www.tarsons.in) containing 1.5 ml of diethyl ether. These tubes were subjected to 200 rpm shaking at 37°C for 4 h; the solvent was then replaced and the process was repeated. Finally after solvent removal, individuals were again dried at 60°C for 48 h and reweighed. Body lipid content was calculated per individual by subtracting the lipid-free dry mass from the initial dry mass per fly.

### Trehalose estimation

For trehalose estimation in three replicates for ten flies of each body color morph of *D. kikkawai*, female flies were individually homogenized in a homogenizer (Labsonic^®^ M; http://www.sartorius.com) with 300 μl Na_2_CO_3_ and incubated at 95°C for 2 h to denature proteins. An aqueous solution of 150 μl acetic acid (1 M) and 600 μl sodium acetate (0.2 M) was mixed with the homogenate. Then, the homogenate was centrifuged (Fresco 21, Thermo Fisher Scientific, PA, USA) at 12,000 rpm (9660× ***g***) for 10 min. For trehalose estimation, aliquots (200 μl) were placed in two different tubes; one was taken as a blank whereas the other was digested with trehalase at 37°C using the Megazyme trehalose assay kit (K-Treh 10/10; http://www.megazyme.com). In this assay, released D-glucose was phosphorylated by hexokinase and ATP to glucose-6-phosphate and ADP, which was further coupled with glucose-6-phosphate dehydrogenase and resulted in the reduction of nicotinamide adenine dinucleotide (NAD). The absorbance by NADH was measured at 340 nm (UV-2450-VIS, Shimadzu Scientific Instruments, Columbia, USA). The pre-existing glucose level in the sample was determined in a control reaction lacking trehalose and subtracted from total glucose concentration.

### Proline estimation

In three replicates of 20 flies of each body color morph, control and fly groups (hardened or acclimated to cold or heat or low humidity pretreatments) were used for proline estimation. Proline content in fly homogenates was determined by the modified method following Bergman and Loxley (1970). In this assay, interference from primary amino acids was eliminated by nitrous acid treatment and the excess nitrous acid was removed by heating with ammonium chloride followed by hydrochloric acid. Interfering materials are also removed by their absorption into the protein-sulphosalicylic acid complex.

For proline content estimation, twenty adult flies were homogenized in 3 ml of sulphosalicylic acid. Following centrifugation, 50 μl of the homogenate was added to 15 μl of freshly prepared 1.25 M sodium nitrite solution and the contents were mixed and kept at room temperature for 20 min. Further, 15 μl of 1.25 M ammonium chloride solution was added and the contents were mixed followed by an addition of 60 μl of concentrated hydrochloric acid. The contents were mixed and heated in a boiling water bath for 20 min. The tubes were cooled and 60 μl of 10 N sodium hydroxide was added. To the resulting solution, we added 200 μl glacial acetic acid and 200 μl of ninhydrin solution in each capped tube. The solutions were then mixed and incubated for 60 min. at 100°C. Following incubation, the samples were extracted with toluene, and absorbance of the aqueous phase was quantified spectrophotometrically at 520 nm and the amount of proline was estimated in reference to a standard curve.

### Treatment and analysis of data

We used Statistica 7 for calculations and illustrations. We calculated morph frequency, i.e. number of female flies of each morph divided by total number of flies of all the three body color morphs collected during each season. Due to lack of significant differences in the frequency of each morph across three years (based on Kruskal–Wallis test), data were pooled for illustration in Fig. 1A. For cold or heat resistance data on wild flies, statistical differences between basal and hardened/acclimated flies of body color morphs were compared with Kruskal–Wallis test. For each trait, data are shown as mean values (±s.e.) based on three replicates of 20 flies. For wild-caught flies, morph specific hardening/acclimation responses were represented as RAC, i.e. trait value of hardened/acclimated flies minus basal trait value, and this difference was divided by basal trait value (see Kellett et al., 2005). For each quantitative trait (resistance to cold or drought or heat), pooled data on three replicates of 20 flies of each morph were checked for normality and equal variance with the help of Statistica 7.0 before analysis through ANOVAs. For each morph, trait and treatment, all the observed values for the three replicates were considered for analysis. Data on developmental plasticity effects for six stress related traits (in morph strains reared at two growth temperatures 15 versus 25°C) were subjected to ANOVA to find variance due to body color morphs, rearing temperatures and their interactions. Finally, for morph strains reared at 15°C, hardening/acclimation-induced changes in three energy metabolites were compared in terms of RAC effects. Based on estimated basal amount of three energy metabolites (proline, trehalose and total body lipids), we calculated energy content per fly (each body color morph) using standard conversion factors (J/mg) following Schmidt-Nielsen (1990). This helped in calculating energy balance after taking into consideration stressor-induced accumulation (+) or utilization (−) of each energy metabolite while comparing effects of heat versus drought acclimation (or cold versus drought acclimation).

## Supplementary Material

Supplementary information
