# Supplementary material for: Developmental and adult acclimation impact cold and drought survival of invasive tropical Drosophila kikkawai
Source: Biol Open. 2021 Jun 8;10(6):bio058527. doi: 10.1242/bio.058527 (PMC8214421; doi:10.1242/bio.058527)
Supplement: Supplementary information [file biolopen-10-058527-s1.pdf]

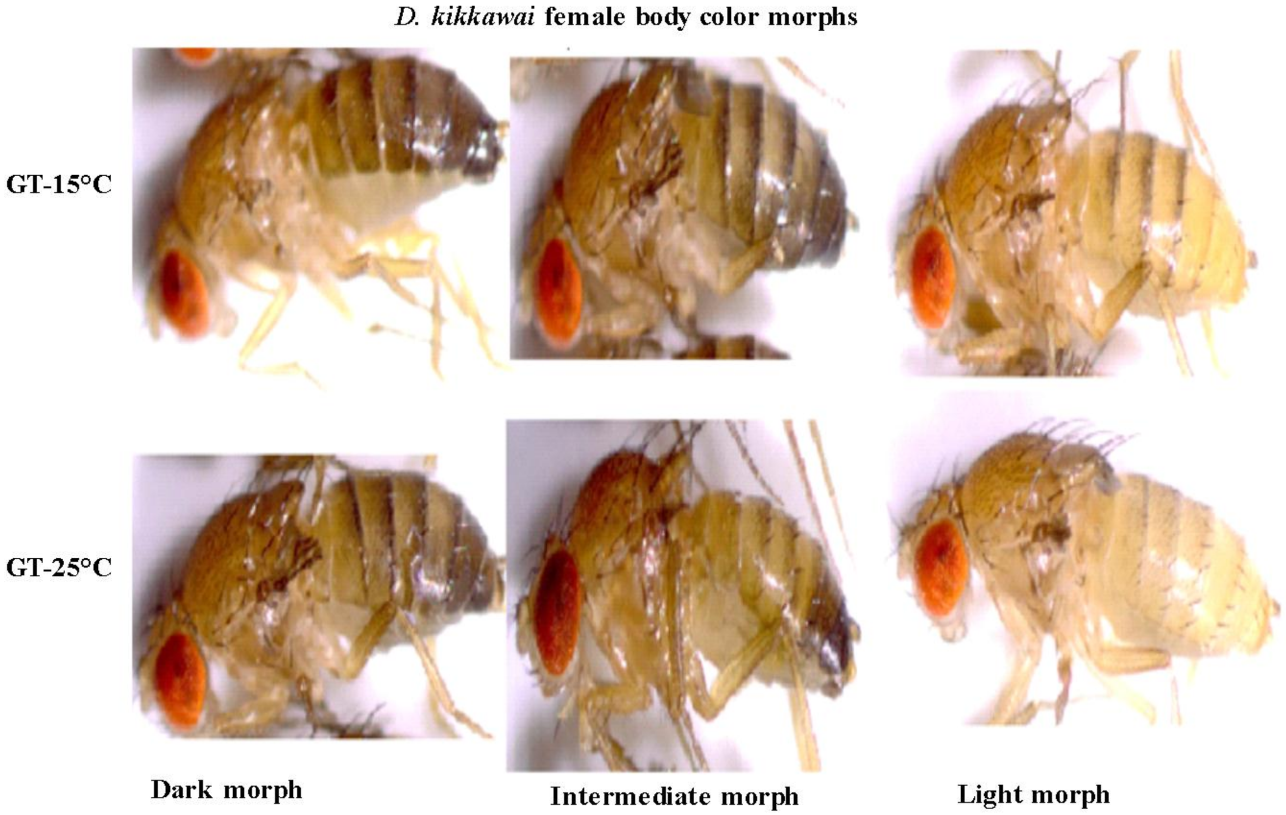

Figure S1. Fly image of three body color morphs (dark, intermediate and light) in females of *Drosophila kikkawai* reared at 15°C (upper row) and at 25°C (lower row).

Biology Open • Supplementary information
